# Quantification of microRNA levels in plasma – Impact of preanalytical and analytical conditions

**DOI:** 10.1371/journal.pone.0201069

**Published:** 2018-07-19

**Authors:** Helle Glud Binderup, Jonna Skov Madsen, Niels Henrik Helweg Heegaard, Kim Houlind, Rikke Fredslund Andersen, Claus Lohman Brasen

**Affiliations:** 1 Biochemistry and Immunology, Lillebaelt Hospital, Kolding and Vejle, Denmark; 2 Department of Regional Health Research, University of Southern Denmark, Kolding, Denmark; 3 Department of Autoimmunology and Biomarkers, Statens Serum Institut, Copenhagen, Denmark; 4 Department of Clinical Biochemistry & Pharmacology, Odense University Hospital, Odense, Denmark; 5 Department of Vascular Surgery, Lillebaelt Hospital, Kolding, Denmark; Case Western Reserve University, UNITED STATES

## Abstract

Numerous studies have reported a potential role for circulating microRNAs as biomarkers in a wide variety of diseases. However, there is a critical reproducibility challenge some of which might be due to differences in preanalytical and/or analytical factors. Thus, in the current study we systematically investigated the impact of selected preanalytical and analytical variables on the measured microRNA levels in plasma. Similar levels of microRNA were found in platelet-poor plasma obtained by dual compared to prolonged single centrifugation. In contrast, poor correlation was observed between measurements in standard plasma compared to platelet-poor plasma. The correlation between quantitative real-time PCR and droplet digital PCR was found to be good, contrary to TaqMan Low Density Array and single TaqMan assays where no correlation could be demonstrated. Dependent on the specific microRNA measured and the normalization strategy used, the intra- and inter-assay variation of quantitative real-time PCR were found to be 4.2–6.8% and 10.5–31.4%, respectively. Using droplet digital PCR the intra-assay variation was 4.4–20.1%, and the inter-assay variation 5.7–26.7%. Plasma preparation and microRNA purification were found to account for 39–73% of the total intra-assay variation, dependent on the microRNA measured and the normalization strategy used. In conclusion, our study highlighted the importance of reporting comprehensive methodological information when publishing, allowing others to perform validation studies where preanalytical and analytical variables as causes for divergent results can be minimized. Furthermore, if microRNAs are to become routinely used diagnostic or prognostic biomarkers, the differences in plasma microRNA levels between health and diseased subjects must exceed the high preanalytical and analytical variability.

## Introduction

MicroRNAs are short (~19–24 nucleotides), single-stranded non-coding RNAs acting as posttranscriptional regulators of gene expression. Cell free microRNAs have been shown to be remarkably stable in blood and other body fluids, as they are released into the extracellular space included in micro vesicles or exosomes or bound to high-density lipoproteins or to the Argonaute2 protein complex [1]. Recently, numerous studies have reported the potential use of circulating microRNAs as diagnostic and prognostic biomarkers in a wide variety of diseases such as cancers [2,3], diabetes [4], autoimmunity [5] and cardiovascular diseases [6]. However, preanalytical and analytical conditions are major sources of variation in and between microRNA studies [7–11], and only a minority of the reported results have been reproduced in other studies. There are many reasons for this but, specifically, our work, and that of others, has shown that the preanalytical centrifugation conditions have a large impact on the measured plasma/serum levels of many microRNAs [7,10,12]. Another important source of variation is microRNA purification. McDonald et. al. estimated that the microRNA purification step accounted for 77–92% of the intra assay variation seen in their microRNA quantification analysis [8]. When considering the analytical conditions, several microRNA detection methods exists, and there is variable agreement between results obtained with the different methods [13]. Furthermore, the choice of normalization strategy may have a significant impact on the reported microRNA levels [14,15].

This study systematically investigates the impact of selected preanalytical and analytical variables, on the measured plasma levels of miR-92a, miR-126 and miR-16. MiR-92a and miR-126 were selected as they have been suggested as circulating biomarkers in cardiovascular diseases [16,17], miR-16 is highly expressed in red blood cells and its plasma levels increase with hemolysis [8], and the three microRNAs are all known to be expressed in platelets [18]. Thus, the agreement between two centrifugation protocols for the preparation of platelet-poor plasma (PPP) for microRNA analysis were investigated, and we compared results obtained with quantitative reverse transcription PCR (RT-qPCR) using microRNA purified from PPP and standard plasma. Furthermore, we investigated the variations in the measured microRNA levels caused by the microRNA-purification step. Finally, we compared three different TaqMan based approaches for microRNA quantification (qPCR (single assays), TaqMan Low Density Arrays (TLDA) and droplet digital PCR (ddPCR)). Normalization was performed using the spike-in cel-miR-39 or the endogenous miR-16, both frequently used by others [15,19,20]. Furthermore, data were normalized using a combination of cel-miR-39 and miR-16, as recently recommended by Poel et. al. [21].

## Materials and methods

### Samples

All samples were venous whole blood samples collected into K2-EDTA containing tubes using a 21 gauge needle (both from Becton-Dickinson, Franklin Lakes, NJ, USA) after a minimum of venous stasis and discarding of the first 3 mL of blood. The blood samples were centrifuged using a Rotina 420R centrifuge (Hettich, Beverly, MA, USA), and all plasma samples were transferred to cryo-tubes and stored at -80°C within 2 hours from blood sampling.

#### Patient samples

Standard plasma (as defined below) and PPP samples from 50 patients with intermittent claudication were collected during June and July 2014 at Lillebaelt Hospital, Kolding, Denmark [22]. All patients gave written informed consent and the study was conducted in agreement with the Helsinki-II declaration and approved by Regional Ethical Committee for the Region of Southern Denmark (S-20140016) and the Danish Data Protection Agency. Standard plasma was obtained from 3 mL EDTA anticoagulated whole blood after a 10 min centrifugation at 2,000 g (acceleration 9, brake 9, room temperature), which is standard procedure in our laboratory when preparing samples for biobanking. PPP was prepared by dual centrifugation. A total of 10 mL EDTA anticoagulated whole blood were centrifuged at 3,000 g for 15 min (acceleration 5, brake 6, temperature 18°C). After centrifugation the plasma phase was carefully transferred to another tube, leaving approximately 1 mL of plasma on top of the buffy coat. The centrifugation step was repeated and again approximately 1 mL was left in the bottom of the tube when the PPP was transferred into cryo-tubes for storage.

#### Samples to compare centrifugation protocols and for precision measurements

A total of 30 EDTA anticoagulated whole blood tubes of 5 mL each were obtained from one healthy volunteer in a single venipuncture. The 30 tubes were labeled with consecutive numbers in the order of draw. From each collection tube PPP was prepared by either dual centrifugation (tubes with odd numbers) or prolonged single centrifugation (tubes with even numbers). The dual centrifugation was performed as described above, except that only 0.5 mL was left behind when transferring the plasma. In the prolonged single centrifugation protocol samples were centrifuged at 3,000 g for 30 min (acceleration 5, brake 6, temperature 18°C), and 1 mL of plasma was left on top of the buffy coat when transferring the PPP to cryo-tubes for storage.

#### MicroRNAs

All experiments were performed with 3 endogenous microRNAs (miR-92a, miR-126 and miR-16) and the exogenous cel-miR-39.

#### Experiments

**Fig 1:**
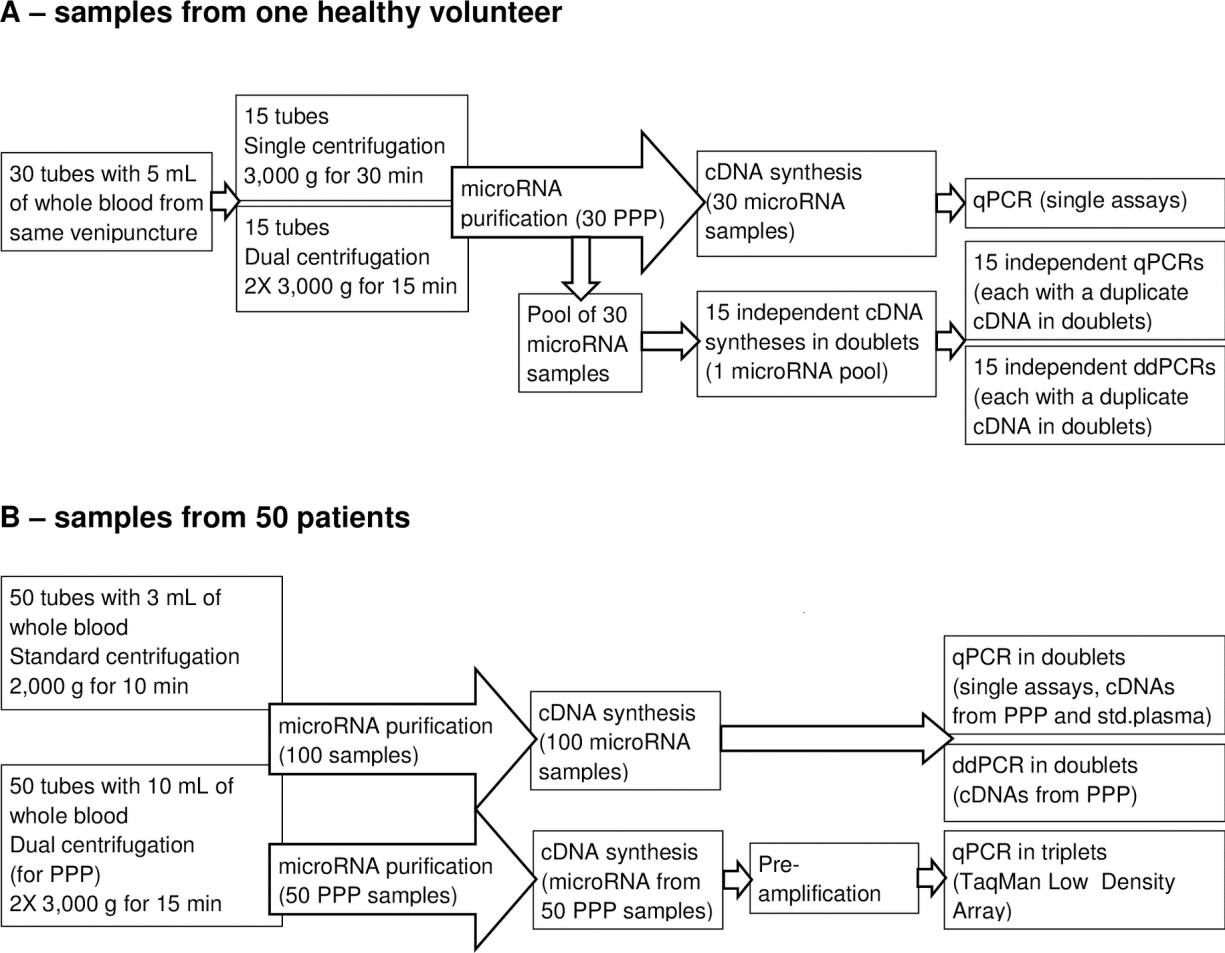
Overview of experiments. Experiments outlined in A) were used to compare dual and prolonged single centrifugation (experiment 1) and to compare qPCR and ddPCR with respect to precision and repeatability (part of experiment 4). Experiments outlined in B) were used to investigate the correlation between qPCR (single assays) and ddPCR (part of experiment 4), correlation between single TaqMan assays and TaqMan Low Density Array (experiment 3) and correlation between TaqMan assays performed with microRNA purified from standard plasma and PPP, respectively (experiment 2).

A schematic overview of the experiments is provided in Fig 1.

**1**)** Dual vs prolonged single centrifugation**

To compare the two centrifugation protocols for the preparation of PPP, we performed RT-qPCR (single assays) with purified microRNA from each of 30 PPPs obtained from a healthy volunteer. As described above, 15 of the samples were prepared by dual centrifugation and 15 samples by prolonged single centrifugation. The procedures for microRNA purification and RT-qPCR are outlined below.

**2**)** PPP vs standard plasma**

Correlation analysis was used to compare measurements obtained with RT-qPCR (single assays) using patient PPP and standard plasma, respectively.

**3**)** Single TaqMan-assays vs TLDA**

Correlation between measurements obtained by single TaqMan-assays and TLDA was assessed for miR-92a and miR-16. Analysis was performed using the 50 patient PPP samples, and following the procedures described below.

**4**)** Comparison of qPCR and ddPCR**

First, the two methods for microRNA quantification were compared with respect to precision and repeatability. A microRNA-pool was prepared from the 30 microRNA samples used above to investigate the centrifugation protocols. In 15 independent analyses on different days, the microRNA-pool was reverse transcribed in doublets, and each of the transcribed cDNAs were assessed, also in doublets, by qPCR (single assays) and ddPCR, respectively. Second, to assess the correlation between measurements obtained by the two methods, microRNA levels in the 50 patient PPP samples were quantified. The procedures for microRNA purification, cDNA synthesis, qPCR and ddPCR are outlined below.

#### MicroRNA purification

MicroRNA was purified from 300 μL of PPP or standard plasma using Nucleospin®miRNA Plasma (Macherey-nagel, Düren, Germany) and according to manufacturer’s protocol. After thawing, all archived standard plasmas were re-centrifuged at 3,000 g for 15 min (room temperature) before the microRNA purification, as we have previously shown, that this will minimize the contamination with microRNA from residual platelets in the plasma [7]. To achieve technical normalization, all samples were spiked with 5 μL of the non-human cel-miR-39 (2.75 × 10^−12^ M) (RiboTask, Odense, Denmark). MicroRNA was eluted with 30 μL of RNAse-free water, and stored at −80°C.

#### RT-qPCR (single assays)

cDNA synthesis was performed using the TaqMan®MicroRNA Reverse Transcription Kit (Applied Biosystems, Foster City, CA, USA) and a RT-primer pool containing microRNA-specific stem-loop primers for miR-92a, miR-126, miR-16 and cel-miR-39 (ThermoFisher assay-IDs: 000431, 002228, 000391 and 000200). The reaction was performed with 2 µL of purified microRNA in a total volume of 15 μL, and the mixture was incubated at 16°C for 30 min, 42°C for 30 min and 85°C for 5 min as recommended by the manufacturer.

Each qPCR contained 1.3 μL transcribed cDNA, 1 μL 20X TaqMan MicroRNA Assay and 10 μL 2X TaqMan Universal PCR Master Mix (Applied Biosystems) in a total volume of 20.3 μL. Each sample was processed in doublets in 40 cycles of 95°C for 15 sec and 60°C for 60 sec using the ABI Prism 7900HT. The mean Ct-values were technically normalized using the exogenous cel-miR-39, and the expression level calculated as 2^-ΔCt^ (ΔCt = Ct_target-miR_−Ct_cel-miR-39_). Furthermore, Ct-values for miR-92a and miR-126 were also normalized using the endogenous miR-16 or the mean of cel-miR-39 and miR-16. A detailed protocol is available at protocols.io (https://doi.org/10.17504/protocols.io.q9edz3e).

#### RT-ddPCR

cDNA synthesis was performed as described above, and the transcribed cDNA was diluted 1:10 with molecular grade H_2_O. Each ddPCR contained 1.1 μL diluted cDNA, 10 μL 2X ddPCR Supermix for Probes (BioRad, Hercules, CA, USA) and 1.0 μL 20X TaqMan MicroRNA Assay (Applied Biosystems) in a total volume of 20 μL. After droplet-generation using the AutoDG (BioRad) the reaction was incubated in 44 cycles of 95°C for 15 sec and 60°C for 60 sec. All samples were run in duplicates, and ddPCR analysis was performed with QX100 Droplet Reader and QuantaSoft Software (BioRad). The mean of the duplicate measurements were normalized to cel-miR-39 or miR-16 by calculating the relative concentration of the target and the reference microRNA. Furthermore, normalization was also performed using the geometric mean of cel-miR-39 and miR-16 as reference. A detailed protocol is available at protocols.io (https://doi.org/10.17504/protocols.io.q8edzte).

#### RT-qPCR using TLDA

cDNA synthesis was performed using the same kits as described for RT-qPCR (single assays), only the RT-primer pool did not contain the primer for miR-126 but included primers for other microRNAs not used in this study. The reaction was performed using 3 μL of purified microRNA in a total volume of 10 μL, and the mixture was incubated in 40 cycles of 16°C for 2 min, 42°C for 1 min and 50°C for 1 sec, followed by 1 cycle of 85°C for 5 min.

As recommended by the manufacturer, a pre-amplification step was performed using 5 μL of the transcribed cDNA in a total reaction volume of 25 μL containing TaqMan®PreAmp Master Mix and Custom TaqMan MIR PreAmp Pool (Applied Biosystems). The pre-amplification mixture was incubated in 14 cycles of 95°C for 15 sec and 60°C in 4 min.

The final quantification was performed using Custom TaqMan®Array MicroRNA Cards (TLDA) from Applied Biosystems. A total of 1.2 μL of the pre-amplification products were mixed with TaqMan®Universal Master Mix II, no UNG and loaded on the array. The samples were assessed in triplets in 40 cycles of 97°C for 30 sec and 59.7°C in 60 sec using the ABI Prism 7900HT, and the mean Ct-values were normalized as described for the single assay RT-qPCR. A detailed protocol is available at protocols.io (https://doi.org/10.17504/protocols.io.q62dzge).

### Statistical analysis

All calculations were performed with normalized data using Stata software, version 15.0 (StataCorp LLC, Texas, USA). Normalization was performed as described above for qPCR and ddPCR using either the spiked-in cel-miR-39, the endogenous miR-16 or a combination of both. The Student t-test was used to compare the microRNA levels obtained with the two centrifugation protocols. A sample size calculation was performed in order to ascertain the number of samples needed to detect a difference of 10% in the mean microRNA level between the two centrifugation methods. All samples were drawn from the same volunteer in a single venipuncture, thus we considered the samples to be paired. Using a standard deviation of 20% of the mean microRNA level (e.g. mean = 10 and SD = 2.0 for miR-92a when normalized to cel-miR-39) and a correlation coefficient of 0.85 we needed 12 samples in each centrifugation protocol (significance level 0.05 and power 0.8).

The Spearman’s rank correlation coefficient (rho) was used to compare the results obtained by RT-qPCR (single assays) to results obtained by RT-qPCR (TLDA) and RT-ddPCR, respectively. Furthermore, results obtained by RT-qPCR using microRNA purified from PPP and standard plasma were compared. P-values below 0.05 were considered as statistical significant.

Precision (inter-assay variability) and repeatability (intra-assay variability) of microRNA measurements were estimated with coefficient of variation [CV = (SD/mean)*100] of the duplicate measurements from 15 independent analyses.

## Results

### Dual vs prolonged single centrifugation for preparation of PPP

When microRNA levels were normalized to cel-miR-39, no significant differences in levels of miR-92a (p = 0.35), miR-126 (p = 0.26) or miR-16 (p = 0.25) between plasmas obtained by the two centrifugation protocols were found. When normalized to the endogenous miR-16 or to the mean of cel-miR-39 and miR-16, we also found no significant differences in levels of miR-92a (p≥0.52), whereas levels of miR-126 were significantly higher in PPP when obtained by dual centrifugation compared to prolonged single centrifugation (P≤0.02),Table 1.

**Table 1:** Comparison of two centrifugation protocols to produce platelet-poor plasma.

	Centrifugation	n	Normalized to cel-miR-39			Normalized to miR-16			Normalized to mean of cel-miR-39 and miR-16		
			*Mean*	*CV (%)*	*P-value*	*Mean*	*CV (%)*	*P-value*	*Mean*	*CV (%)*	*P-value*
miR-92a	*Single*	15	9.9	19.5	0.35	0.095	11.3	0.83	0.97	13.1	0.52
	*Dual*	15	9.2	21.1		0.096	9.5		0.94	13.3	
miR-126	*Single*	15	0.28	10.9	0.26	0.0027	14.0	0.01	0.027	9.2	0.02
	*Dual*	15	0.30	17.3		0.0031	14.2		0.030	12.5	
miR-16	*Single*	15	105	19.0	0.25						
	*Dual*	15	96	19.4							

30 tubes of EDTA-anticoagulated whole blood were drawn from a peripheral vein of a healthy volunteer. From each tube platelet-poor plasma (PPP) was prepared by either dual centrifugation or a prolonged single step centrifugation. MicroRNA-levels in each PPP were measured using RT-qPCR (single assays) and normalized to either cel-miR-39, miR-16 or the mean of cel-miR-39 and miR-16. For each centrifugation protocol and with all normalization strategies, the mean relative microRNA level and the coefficient of variation (CV) are provided. P-values (t-test) are shown for the comparison of the mean of the two centrifugation protocols.

Coefficients of variation were found to be high with both centrifugation strategies and higher when microRNA levels were normalized to cel-miR-39 (10.9–21.1%) as compared to normalization using miR-16 (9.5–14.2%) or a combination of cel-miR-39 and miR-16 (9.2–13.3%), Table 1.

**Fig 2:**
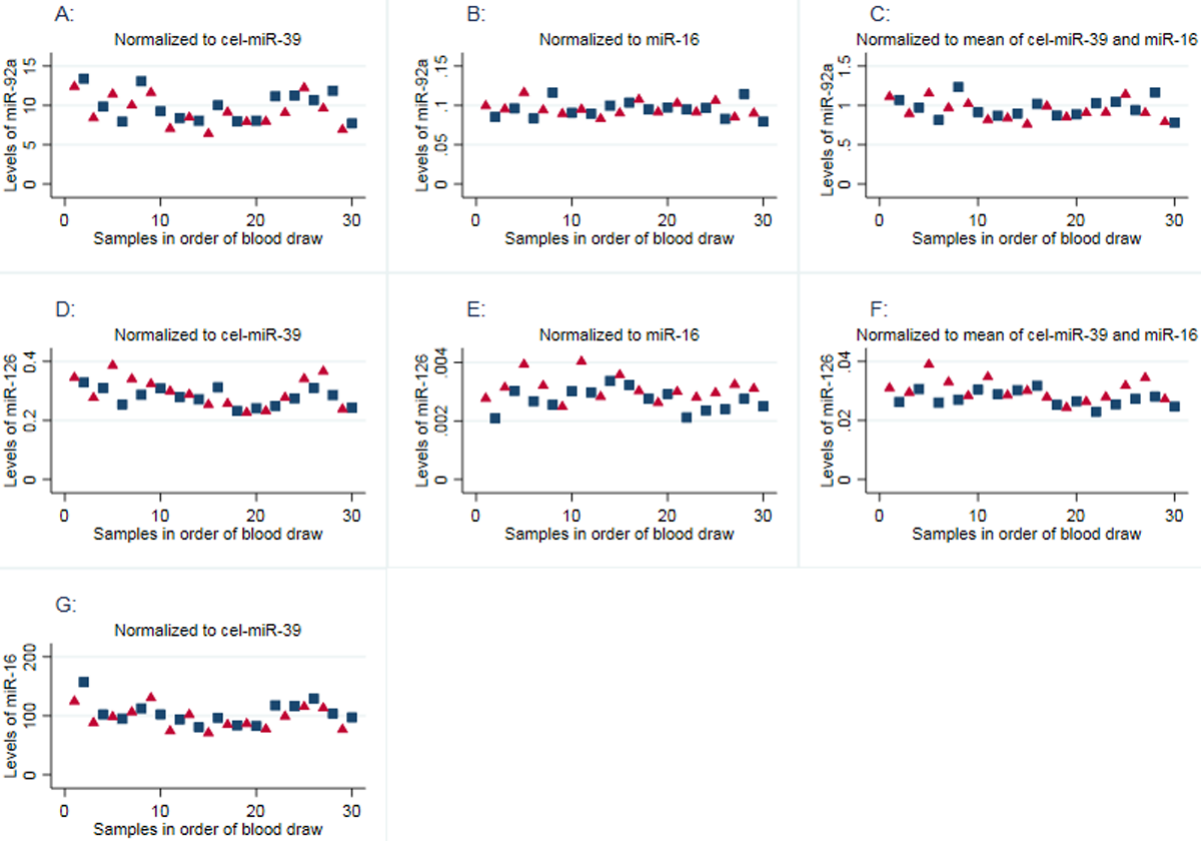
Relative microRNA levels and order of blood draw. Plots showing the relative microRNA levels in 30 PPP samples from a healthy volunteer in order of blood draw. Samples with odd numbers were prepared by dual centrifugation (red triangles) and samples with even numbers by a prolonged single centrifugation (blue squares). MicroRNA-levels were normalized to cel-miR-39 (plots A, D and G), miR-16 (plots B and E) or the mean of cel-miR-39 and miR-16 (plots C and F). In all cases, we found no significant differences (t-test) in the mean microRNA-levels between the first 10 tubes and the last 10 tubes drawn (p>0.05).

When plotting the microRNA levels of the 30 PPPs, we see no clear pattern to indicate that the measured microRNA levels are influenced by the drawing order, Fig 2. Furthermore, when comparing microRNA-levels in the first 10 tubes and the last 10 tubes drawn, we found no significant differences in the mean levels of miR-92a (p = 0.31), miR-16 (p = 0.41) and miR-126 (p = 0.08) when normalized to cel-miR-39, in levels of miR-92a (p = 0.66) and miR-126 (p = 0.41) when normalized to miR-16, or in levels of miR-92a (p = 0.36) and miR-126 (p = 0.14) when a combination of cel-miR-39 and miR-16 were used for normalization.

### Platelet-poor plasma vs standard plasma

When using cel-miR-39 as a mean of normalization, we found no correlation between measurements obtained with PPP and standard plasma as starting material (rho≤0.28 and p≥0.05). When microRNA levels were normalized to miR-16, we found intermediate correlation between measurements obtained with the two starting materials, rho = 0.42 (p = 0.0024) for miR-92a and rho = 0.48 (p = 0.0004) for miR-126. An intermediate correlation was also observed, when miR-126 levels were normalized to a combination of cel-miR-39 and miR-16 (rho = 0.47, p = 0.0005), whereas with this normalization strategy we found no correlation for miR-92a (rho = 0.02, p = 0.88), (Parts D-I and K of S1 Fig).

### Single TaqMan-assays vs TLDA

We assessed the levels of miR-92a in the 50 patient PPP samples using single TaqMan-assays and TLDA, and found no correlation between measurements from the two methods regardless of the normalization strategi used (all rho≤0.23 and p≥0.11). When levels of miR-16 were normalized to cel-miR-39, correlation between the two methods were found to be intermediate (rho = 0.47, p = 0.0005), (Parts A-C and J of S1 Fig).

### qPCR vs ddPCR

We found the inter-assay precision of the two methods to be similar, but with a tendency to be slightly lower with ddPCR as compared to qPCR. Contrary, we found the repeatability (intra-assay precision) to be significantly lower with qPCR as compared to ddPCR, except for miR-92a when normalized to miR-16 or to the mean of cel-miR-39 and miR-16. Results are outlined in Table 2.

**Table 2:** Precision and repeatability of qPCR and ddPCR.

			Normalized to cel-miR-39		Normalized to miR-16		Normalized to mean of cel-miR-39 and miR-16	
			*Mean*	*CV (%)*	*Mean*	*CV (%)*	*Mean*	*CV (%)*
miR-92a	*qPCR*	*Precision*	16.9	31.4	0.16	22.2	1.62	26.5
		*Repeatability*		6.8		6.3		6.0
	*ddPCR*	*Precision*	5.8	26.7	0.22	21.6	1.13	23.7
		*Repeatability*		13.2		4.4		7.8
miR-126	*qPCR*	*Precision*	0.29	15.6	0.0028	10.5	0.028	11.1
		*Repeatability*		5.1		4.8		4.2
	*ddPCR*	*Precision*	0.78	12.4	0.029	5.7	0.15	7.0
		*Repeatability*		20.1		13.4		15.9
miR-16	*qPCR*	*Precision*	107	13.2				
		*Repeatability*		5.2				
	*ddPCR*	*Precision*	26.5	12.4				
		*Repeatability*		11.3				

A microRNA pool was reverse transcribed in doublets in 15 independent cDNA syntheses on different days. The transcribed cDNAs were assessed in doublets in 15 independent qPCR and ddPCR. The 15 duplicate measurements were used to calculate the precision (inter-assay variation) and repeatability (intra-assay variation) of the two methods for microRNA quantification. The table shows for each method the mean of all 30 measurements and the coefficient of variation (CV).

**Fig 3:**
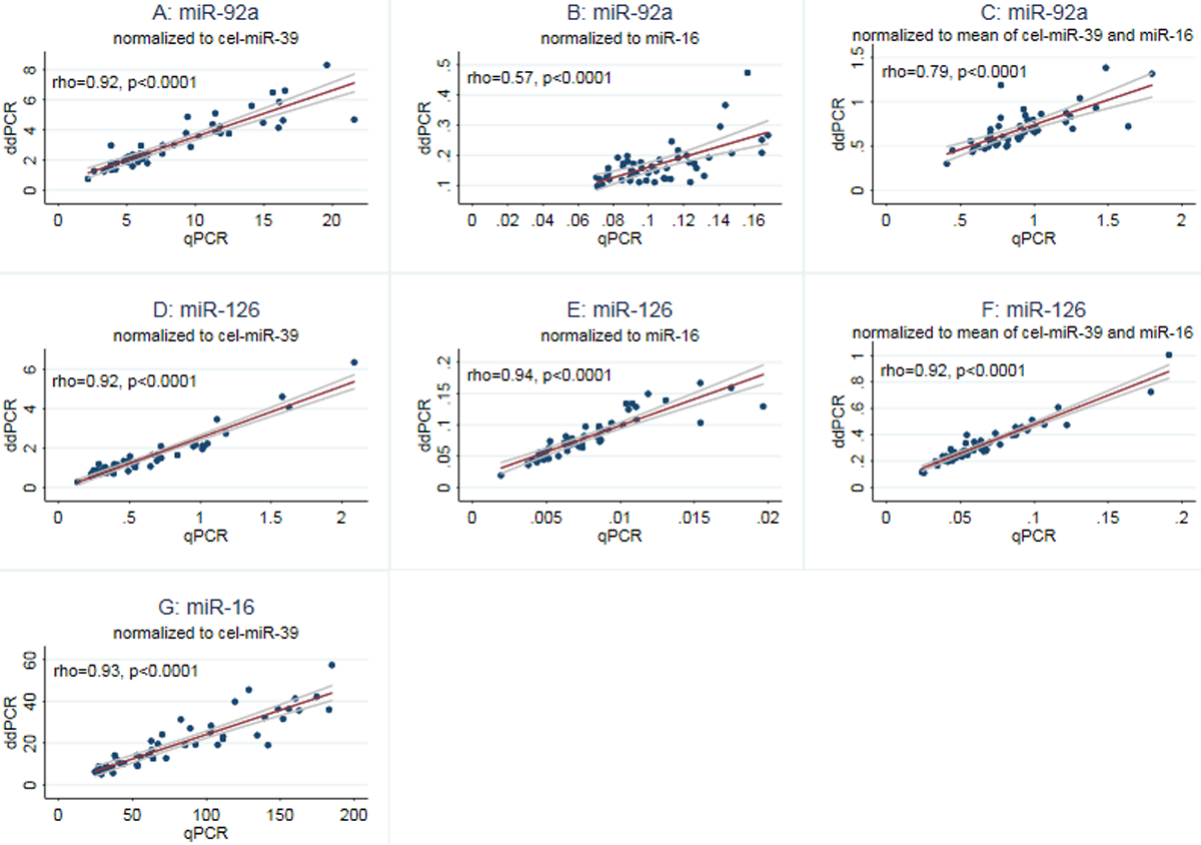
Correlation of measurements obtained by qPCR and ddPCR. Plots showing the correlation (with 95% confidence interval) between microRNA quantification performed by single TaqMan assays (= qPCR) and ddPCR. In plot A, D and G the target microRNAs are normalized to cel-miR-39, in plot B and E miR-16 was used for normalization, and in plot C and F data were normalized to the mean of cel-miR-39 and miR-16.

Correlation of measurements obtained by qPCR and ddPCR were found to be very good when microRNA levels were normalized to cel-miR-39 (rho≥0.92 and p<0.0001 for all microRNAs measured). When microRNA levels were normalized to miR-16 or to the mean of cel-miR-39 and miR-16 the correlation between the two methods was still very good for miR-126 (rho≥0.92, p<0.0001), but only moderate for miR-92a (rho = 0.57–0.79, p<0.0001), Fig 3.

### Variation due to centrifugation and microRNA-purification

To estimate the variation in the measured microRNA-levels due to the centrifugation and microRNA purification steps, we compare the CVs obtained when investigating the centrifugation protocols (Table 1) to the intra-assay precision for qPCR (Table 2). In Table 1 the CVs are parallels to the intra-assay precision of the total procedures from whole blood to result, whereas the intra-assay CVs in Table 2 do not include the centrifugation and purification steps. For each microRNA, the percentage of the intra-assay variation caused by centrifugation and microRNA purification was calculated as (CV_total_−CV_RT-qPCR_)/CV_total_, where CV_total_ is the mean of the CVs estimated for the dual and prolonged single centrifugations (Table 1) and CV_RT-qPCR_ is the intra-assay precision of qPCR (Table 2). The centrifugation and microRNA purification steps were found to account for 64–73% of the intra-assay variation when using cel-miR-39 for data normalization, 39–66% when data were normalized to miR-16, and 55–61% when normalized to a combination of cel-miR-39 and miR-16.

## Discussion

MicroRNAs are promising biomarkers for diagnostics and prognostics in a wide variety of diseases, but there is an urgent need for standardization of the preanalytical, analytical and postanalytical conditions for their quantification. Here we compared different centrifugation protocols for the preparation of plasma samples for microRNA analysis, determined the variations in microRNA levels caused by microRNA-purification, and compared three different TaqMan-based approaches for microRNA quantification (single qPCR assays, TLDA and ddPCR).

We compared microRNA-levels in PPP obtained by dual and prolonged single centrifugation. When microRNA levels were technically normalized to cel-miR-39 we found no significant differences between results obtained using the two centrifugation protocols, and we also found no differences in miR-92a levels when normalized to the endogenous miR-16 or to a combination of the two reference microRNAs. In contrast, when normalized to miR-16 or to the two reference microRNA in combination, we found miR-126 levels to be significantly higher in PPP obtained by dual centrifugation. Nevertheless, in PPP the level of miR-16 was found to be 256 fold (~8 ct-values) higher than the level of miR-126, which makes miR-16 less suitable as a mean of normalization of miR-126 levels. By way of comparison, there were 8–16 fold (3–4 ct-values) differences in levels of miR-16 and miR-92a, and only 4 fold (~2 ct-values) differences in levels of cel-miR-39 and miR-126. Coefficient of variation was found to be high, and largely independent of the centrifugation protocol used. Furthermore, though one might speculate that due to platelet activation or hemolysis, microRNA levels in the blood collection tubes may increase over the time course of the blood sampling procedure, when using a minimal of stasis, we found no significant impact on measured microRNA-levels of the order of draw. To our knowledge this is the first study to provide evidence for the possibility to use a single centrifugation step for preparation of PPP, and that the order of blood draw has no significant impact on the results of microRNA analyses.

We have previously shown that the plasma levels of a number of microRNAs are higher in standard plasma compared to PPP [7]. In the present study we investigated whether the relative differences between patient-samples are the same in the two types of plasma. We found no correlation between results obtained using standard plasma and PPP as starting material when microRNA-levels were normalized to cel-miR-39 or to a combination of cel-miR-39 and miR-16, and only intermediate correlation when data were normalized to miR-16. Therefore, it is very important to provide a comprehensive description of the type of material used and how it was processed, when publishing results from microRNA studies.

MicroRNAs have been found to be promising biomarkers for a wide variety of diseases, but in many cases the results are never validated by others, which in part may be due to the differences in the microRNA-platform used [23]. We compared results obtained by single TaqMan assays and TLDA, and found no correlation between miR-92a measurements, and only intermediate correlation between miR-16 measurements. TLDA and single TaqMan assays use the same methodology, and thus the lack of correlation between measurements must be based on differences in preanalytical and analytical conditions. The pre-amplification step used in the TLDA protocol, will enhance differences due to microRNA purification, pipetting of the spike, pipetting reagents for cDNA synthesis and variations in amplification efficiencies of microRNA assays. The large difference in reaction volume used, 1 μL for TLDA and 20.3 μL for the single assay, may also influence the efficiency of the PCR-reaction. Several other authors have presented discrepancies in results with TLDA and single TaqMan assays. Jorge et. al. used single TaqMan assays to validate their findings from a TLDA-analysis, and found that the two methods were reproducible and comparable for 7 of the 16 microRNAs analyzed, and in agreement with our study they could not reproduce the TLDA findings for miR-92a in the qPCR analysis [24]. Other studies also found, that differences in microRNA levels between groups as detected by TLDA analyses, can only partly be reproduced in a validation cohort by the use of single TaqMan assays [25–27]. In the 4 studies mentioned, the lack of agreement between the two platforms may be due to differences in the patient cohorts used for screening and validation. Darvasi et. al. used the same patient samples for TLDA analyses and analyses using single TaqMan assays, and were only able to validate the TLDA findings for 76.2% of the microRNAs investigated [28]. In general caution should be taken when comparing even highly similar methods and findings should be validated using identical methods.

Campomenosi et. al. used ddPCR (EvaGreen) and qPCR (SYBR Green) to perform absolute quantification of 3 microRNAs in serum, and found very good correlation between results obtained by the two techniques (R-square = 0.95 and p<0.0001 for all microRNAs investigated) [29]. In the present study, we used the probe based TaqMan assays to perform a relative quantification of microRNA levels in plasma, and found the same good correlation between ddPCR and qPCR when microRNA levels were normalized to cel-miR-39. When normalizing to miR-16 or to a combination of cel-miR-39 and miR-16, the correlation between the two techniques was only moderate for miR-92a.

In the study by Campomenosi et. al., aliquots of cDNA-samples were used to compare the inter-assay precision of microRNA measurements obtained with ddPCR and qPCR. They found in microRNA-126 analysis the mean CV to be 7.94% and 7.39%, respectively [29]. Other studies using ddPCR for absolute quantification of microRNA have measured the inter-assay precision of the method to be 20% (miR-141) [30] and 13.4% (cel-miR-39) [31]. These results are in line with our findings, and the findings of others, when adjusting for differences in study design. We used aliquots of a microRNA-pool to perform 15 independent duplicate cDNA syntheses, and analyzed each duplicate cDNA in an independent qPCR and ddPCR. Therefore, our analyses include variations due to the reverse transcription, and thus we are expected to find higher CVs as compared to Campomenosi et. al. Tomasetti et. al. used aliquots of serum and TaqMan qPCR analysis to measure the relative levels of miR-126 normalized to cel-miR-39, and found the intra-assay and inter-assay precision to be 16% and 11%, respectively. Furthermore, they found that the CVs were lower when microRNA levels were normalized using a combination of cel-miR-39 and the endogenous U6 [32]. In the present study we found lower CVs when normalizing to the endogenous miR-16 or to a combination of cel-miR-39 and miR-16, but as mentioned above miR-16 is not always a good choice of reference microRNA, due to its high levels in PPP.

We found that together, the centrifugation and purification steps in our microRNA analysis accounted for 64–73% of the intra-assay variation when using cel-miR-39 for data normalization, 39–66% when data were normalized to miR-16 and 55–61% when a combination of the two reference microRNAs were used. In line with this, McDonald et. al. found RNA-extraction to account for 77–92% of the variation in their microRNA-analyses [8].

The strengths of our study are that the same set of samples was used to compare the different platforms, that the same patient cohort was used to compare PPP to standard plasma, and that all analyses were performed by the same scientist. Furthermore, the intra- and inter-assay variations were estimated using results from 15 independent duplicate analyses each performed with purified microRNA as starting material. The relatively low number of microRNA examined may be a limitation, and the choice of references for normalization may influence the results obtained, though the same reference miRNAs were used in all the methods compared.

In conclusion, our results highlight the importance of a comprehensive description of the preanalytical and analytical factors used, when publishing results from microRNA studies, in order to allow for others to perform the exact same experiments in validation studies.

Furthermore, when conclusions are made regarding observed differences in microRNA-levels between healthy and diseased subjects or between groups of patients, the high intra- and inter-assay variability of the analyses must be taken in to account. If microRNAs are to become routinely used biomarkers in a clinical setting, their quantification must be reproducible, and differences in plasma levels must be of a magnitude large enough to overcome the impact of preanalytical and analytical variability.

## Supporting information

S1 Fig Correlation. Parts A-C and J shows the correlation between microRNA measurements obtained by single TaqMan assays (single qPCR) and TaqMan Low Density Array (TLDA). Parts D-I and K shows the correlation between microRNA measurements in PPP and standard plasma (single TaqMan assays). In parts A, D, G, J and K the target miRNAs are normalized to cel-miR-39, in parts B, E and H miRNA-16 was used for normalization, and in parts C, F and I data are normalized to the mean of cel-miR-39 and miR-16.
(TIF)

S1 Data Ct-values of all experiments. (XLSX)
