# Supplementary figures and images for: Quantification of microRNA levels in plasma – Impact of preanalytical and analytical conditions
Source: PLoS One. 2018 Jul 19;13(7):e0201069. doi: 10.1371/journal.pone.0201069 (PMC6053236; doi:10.1371/journal.pone.0201069)

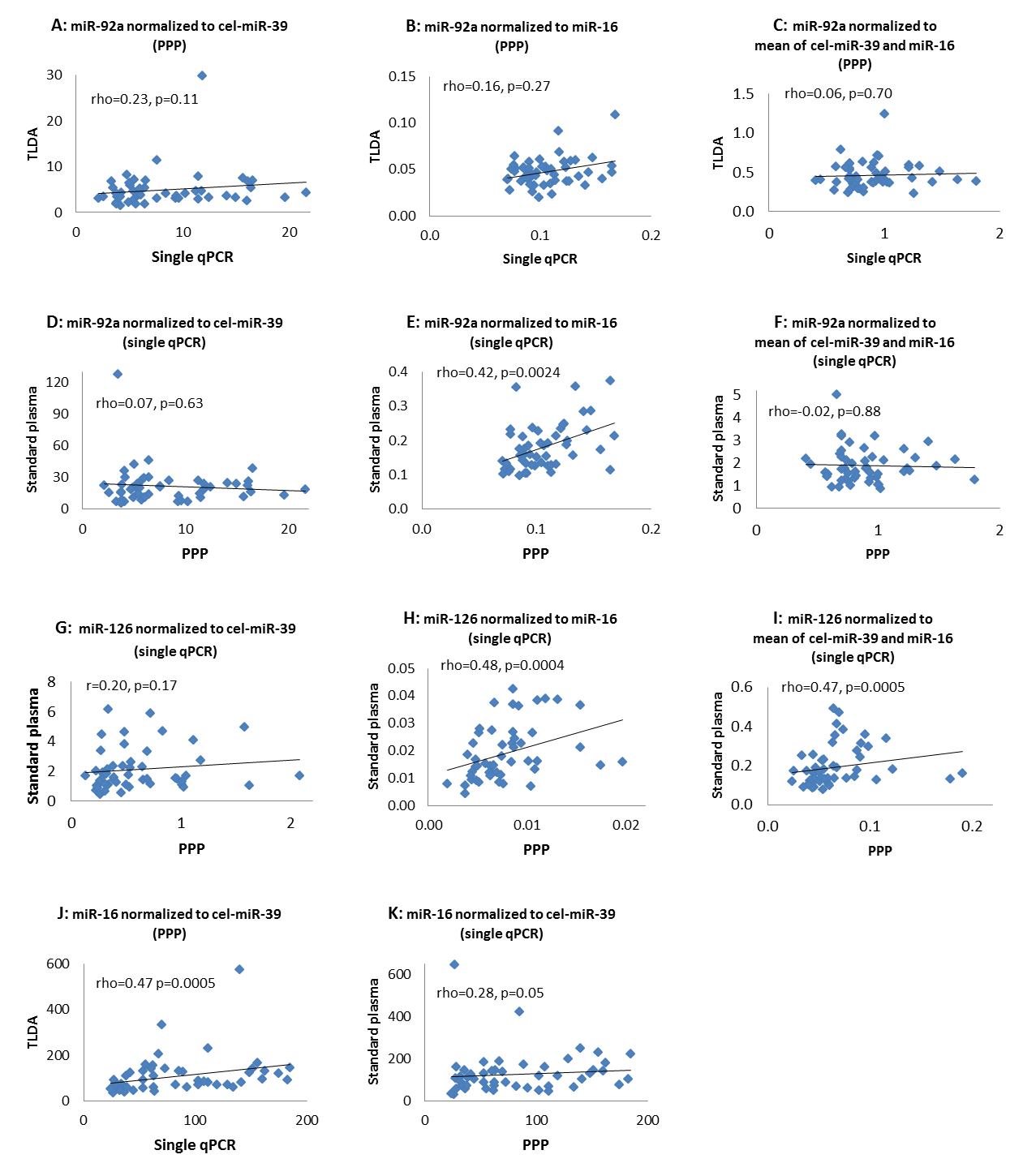

Supplement: S1 Fig — Parts A-C and J shows the correlation between microRNA measurements obtained by single TaqMan assays (single qPCR) and TaqMan Low Density Array (TLDA). Parts D-I and K shows the correlation between microRNA measurements in PPP and standard plasma (single TaqMan assays). In parts A, D, G, J and K the target miRNAs are normalized to cel-miR-39, in parts B, E and H miRNA-16 was used for normalization, and in parts C, F and I data are normalized to the mean of cel-miR-39 and miR-16. (TIF) [file pone.0201069.s001.tif]
